# New tools for calibrating diffraction setups

**DOI:** 10.1107/S1600577520000776

**Published:** 2020-02-20

**Authors:** J. Kieffer, V. Valls, N. Blanc, C. Hennig

**Affiliations:** a ESRF – The European Synchrotron, CS40220, 38043 Grenoble Cedex 9, France; b University Grenoble Alpes, Grenoble INP, 38000 Grenoble, France; c CNRS, Institut NEEL, 38000 Grenoble, France; d Helmholtz-Zentrum Dresden-Rossendorf, Institute of Resource Ecology, Bautzner Landstrasse 400, 01328 Dresden, Germany; e The Rossendorf Beamline at ESRF, BP 220, 38043 Grenoble Cedex 9, France

**Keywords:** powder diffraction, geometry calibration, goniometers, moving detectors, translation tables, pair distribution function

## Abstract

New calibration tools in the *pyFAI* suite for processing scattering experiments acquired with area detectors are presented. These include a new graphical user interface for calibrating the detector position in a scattering experiment performed with a fixed large area detector, as well as a library to be used in *Jupyter notebooks* for calibrating the motion of a detector on a goniometer arm (or any other moving table) to perform diffraction experiments.

## Abstract 2

New tools to calibrate different types of scattering experiments suitable for static and moving detectors are presented.

## 1. Introduction   

According to an internal survey of the beamline scientists at the European Synchrotron (ESRF) in 2016, the factor limiting user productivity at diffraction beamlines is the calibration of the experimental setup before the raw data can be reduced to analysable data. The calibration issue is currently worked around by providing users with the proper geometry description or even the reduced data; however, this prevents re-processing at home institutes and also re-interpretation of data, which is needed as a part of the open-data initiative (Wilkinson *et al.*, 2016 ▸).

At X-ray facilities such as synchrotons, large area detectors are preferred for gathering the maximum number of photons in scattering experiments (powder diffraction, small-angle scattering, *etc.*). Using a fixed-position setup combined with the speed of modern detectors (up to a few kHz), it is easy to follow chemical reactions or other physical processes *in situ*. Even with fixed geometries, determining the detector position is still an issue for the majority of users. Hence, a new graphical user interface has been developed for the Python fast azimuthal integration library *pyFAI* (Kieffer *et al.*, 2019 ▸), focusing on the user experience to grant them autonomy in data analysis once they have returned to their home institutes.

Laboratory source diffractometers are commonly equipped with (small) area detectors mounted on moveable goniometer arms for powder diffraction (for example, the Rigaku HyPix-3000 detector). Detectors at synchrotrons are often mounted on goniometer or translation tables to provide the degrees of freedom (hereafter DoFs) needed to align the beamline, although those DoFs are rarely used for data acquisition. One counter-example is reported by Gao *et al.* (2016 ▸), where the beamline is equipped with a moving strip-detector, the Mythen detector (1D) from Dectris, but the Mythen is not an area detector.

Pair distribution function (PDF) analysis typically requires very large area detectors and higher energies to be able to cover the needed high *q*-range in one single frame (Chupas *et al.*, 2003 ▸). When speed is not critical, PDF experiments can be performed with small area detectors mounted on a motorized arm, and moved in front of the sample during data acquisition to cover a larger solid angle. A new *pyFAI* module which handles goniometers and moving detectors is also presented. It offers the ability to exploit the positioning system of the detector (often readily available on beamlines) to acquire powder diffraction or PDF data on a larger *q*-range with no additional cost or investment.

The *pyFAI* library (Ashiotis *et al.*, 2015 ▸) is first briefly introduced, then the merging of multiple diffraction images acquired at different positions is demonstrated, as shown by Kieffer & Wright (2013 ▸). After summarizing how calibration works in *pyFAI*, the new graphical user interface is presented. Finally, the procedure on how to calibrate the absolute position of every single pixel in the detector when mounted on a goniometer (or on a translation stage) as a function of motor positions is described.

## 2. The Python fast azimuthal integration library   

*pyFAI* is a Python (van Rossum, 1989 ▸) library used to transform 2D diffraction images into 1D powder diffraction patterns by re-binning the pixel positions in polar coordinates. The radial units are typically the scattering angle 2θ or the momentum transfer *q* = 4πsin(θ)/λ. *pyFAI* also provides additional tools to calibrate the detector position, *i.e.* to determine its location in space by means of Debye–Scherrer conical rings resulting from the intersection of the diffracted X-ray beam with the detector surface. The observed rings are deformed into ellipses when the detector is planar but slightly inclined. The azimuthal integration is performed in two steps. The first is a pixel-wise transformation corresponding to the image correction,
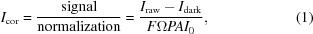
where *I*_raw_ is the raw signal of the detector, *I*_dark_ is the dark current image (it may also be the background image for certain experiments), *F* is the factor accounting for the flat-field correction, Ω is the solid angle subtended by a given pixel, *P* is the polarization correction term and *A* represents the detector’s apparent efficiency due to the incidence angle of the photon on the detector (for integrating detectors, high-energy photons with larger incidence angles see larger sensor thickness, and thus have a higher detection probability). *I*_raw_ may be normalized by the incoming flux *I*_0_, which is independent of the pixel position. The numerator of equation (1) will hereafter be referred to as the ‘signal’, whilst the denominator will be referred as ‘normalization’. The ‘variance’ associated with the signal of every single pixel can be estimated from statistical distributions assuming, for example, that the detector has a Poissonian behaviour. This variance needs to be scaled in a similar way when considering error propagation.

The second step is the re-binning of the data, which is carried out using histograms of the radial positions, weighted by either the signal or normalization as described by Kieffer & Ashiotis (2014 ▸). This histogram sums the signal (resp. normalization) for all pixels having a radius corresponding to a given radial bin (this set of pixels is denoted bin_r_). The intensity averaged over all pixels located at a given radial value is then simply the ratio of the two histograms which bin corresponds to the radius of interest [equation (2)][Disp-formula fd2]. The propagated error (standard error of the mean) is given in equation (3),
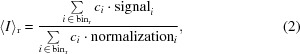


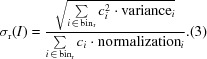
In equations (2) and (3), *c*_*i*_ is used to describe pixel splitting and corresponds to the fraction of the pixel area falling into the specific histogram bin (0 ≤ *c*_*i*_ ≤ 1). The multiple pixel-splitting schemes available in *pyFAI*, called full-splitting, bounding-box-splitting and no-splitting, have been described by Ashiotis *et al.* (2015 ▸).

The integration scheme presented in equation (2) is an improvement over former versions of *pyFAI* (version <0.18), which used equation (4) where the error propagation was not properly performed as pixels with smaller normalization factors were over-represented in the associated errors,
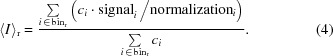



## 3. Azimuthal integration of multiple frames taken at multiple geometries   

The *pyFAI* integration scheme of multiple images taken at various positions was first reported by Kieffer & Wright (2013 ▸). The procedure is conceptually similar to the integration on a single image, except that all histograms need to be calculated over the very same grid (bin positions) to allow histograms from all images to be summed together, as described in equation (5),
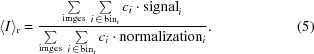
The normalization for solid-angle correction, Ω, has to be performed using an absolute solid-angle reference system as different geometries may have very different sample-to-detector distances. This is different from the integration in single-frame mode, where *pyFAI* uses the solid angle relative to the point of normal incidence (PONI). Hence, integrated intensities in multi-geometry mode are orders of magnitude larger than usual, the scale factor being the solid angle of one pixel at the PONI, *i.e.* dist^2^/(pixel_1_ × pixel_2_). Errors are propagated in an similar way to equation (3), taking into account the absolute solid-angle normalization.

## 4. Calibration of the detector position   

### 4.1. Principle of the calibration using a Debye–Scherrer diffraction image   

The calibration of a detector position is performed using several Debye–Scherrer rings collected from a reference powder called the *calibrant*. The rings are extracted automatically from the image (by subtracting a smoothed version of the image) and control points are placed at the local maxima on the rings. The geometry of the experiment is obtained by refining geometric parameters using a least-square fitting of the 2θ values calculated for the different control points.

**Figure 1:**
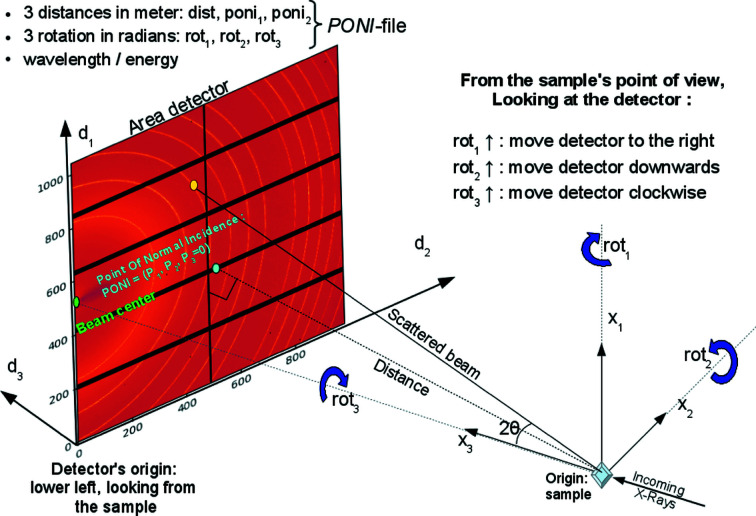
Geometry used in *pyFAI*.

Intuitively, the easiest geometry to perform the calibration is built upon the beam-centre definition, which is the intersection of the direct beam with the detector. This geometry was first introduced in *FIT2D* (Hammersley *et al.*, 1996 ▸) and re-used in many software packages such as *GSAS-II* (Toby & Von Dreele, 2013 ▸) and *DAWN* (Filik *et al.*, 2017 ▸) which also offer user-friendly interfaces. The *FIT2D* geometry reaches its limits when the detector is heavily tilted, and it is not even possible to describe a detector mounted parallel to the beam (which is sometimes used on laboratory sources in reflection geometry). The geometry used in *pyFAI* is based on the definition of the PONI (Fig. 1 ▸), which is the orthogonal projection of the sample position (the origin in *pyFAI*) on the detector plane (or the plane *z* = *d*_3_ = 0 when the detector is non-planar and *z* varies from pixel to pixel). This geometry is inspired by *SPD* (Boesecke, 2007 ▸) and is capable of describing any detector position in space. It is worth noting that the PONI is co-located with the beam centre when the detector is not tilted, *i.e.* mounted orthogonal to the direct beam. Moreover, the PONI is more likely than the beam centre to be located within the detector surface when the detector is heavily tilted (since the detector gathers more photons when facing the sample). Some geometry conversion tools are provided by *pyFAI* and documented by Detlefs & Kieffer (2019 ▸).

The geometrical parameters to be refined are the following: (i) *dist*: the distance (in metres) from the sample position to the PONI. (ii) *poni*_1_ and *poni*_2_: space coordinates of the PONI (in metres) within the detector plane (*z* = *d*_3_ = 0) along the slow- and fast-reading dimension of the detector image (1 and 2 usually refer to the row and the column axis, *i.e. y* and *x*, respectively). (iii) *rot*_1_, *rot*_2_ and *rot*_3_: the rotation angles (expressed in radians) of the detector placed at the proper distance from the sample, with respect to the three axes of the laboratory reference system. The detector is first rotated around the vertical axis (*rot*_1_), then around the horizontal axis (*rot*_2_) and finally around the incoming beam direction (*rot*_3_). In a more mathematical way, this gives
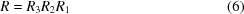
where
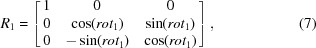


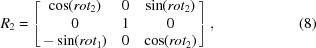


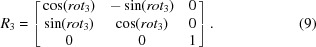
It is worth mentioning that rotations *R*_1_ and *R*_2_ are left-handed, while *R*_3_ is right-handed, which is a legacy from previous versions of *pyFAI*.

The strength of this geometry parameterization is that it describes any detector position in space. The drawback is that some parameters are correlated or not optimized:

(i) *dist-wavelength*: reducing the wavelength is nearly equivalent to increasing the distance unless the diffraction angle is large (2θ > 30°). It is advisable to fix one of these two variables unless the data quality are good enough and the scattering range is very large. This is an intrinsic limitation of the single-image approach.

(ii) *rot*_1_-*poni*_2_ and *rot*_2_-*poni*_1_: as small rotations can be mistaken for larger translations. Fixing one of the two decreases the uncertainty of the other by several orders of magnitude. The user interface offers a ‘SAXS constrains’ button to lock the detector normal to the direct beam.

(iii) *rot*_3_: cannot be refined from Debye–Scherrer cones as they are, by definition, invariant by rotation around the incoming beam. The *rot*_3_ parameter can be assessed using an anisotropic contribution such as the polarization effect or some textures in the sample. This *rot*_3_ parameter is used to describe the azimuthal rotation of the experimental setup or of the sample, for example, when calculating pole figures.

Those calibration parameters containing the geometry of the experimental setup are saved in text files with a .poni extension, also containing the detector definition and the wavelength. Such a file can subsequently be loaded into an *azimuthal integrator* object, ready to perform the azimuthal regrouping of images coming from the detector.

### 4.2. Graphical user interface   

A semi-graphical calibration tool has been available as part of *pyFAI* since the origin of the project (Ashiotis *et al.*, 2015 ▸), but this tool was considered too difficult to use by inexperienced users. Thus, many groups have developed their own graphical user interface on top of *pyFAI*, often optimized for their specific setup or experiment: *Dioptas* (Prescher & Prakapenka, 2015 ▸) focusing on high-pressure experiments, *Dpdak* (Benecke *et al.*, 2014 ▸) for online SAXS, *NanoPeakCell* (Coquelle *et al.*, 2015 ▸) for serial crystallography, *PySAXS* (Taché *et al.*, 2015 ▸), *WiSAXS* or *xPDFsuite* (Yang *et al.*, 2014 ▸) to cite a few.

**Figure 2:**
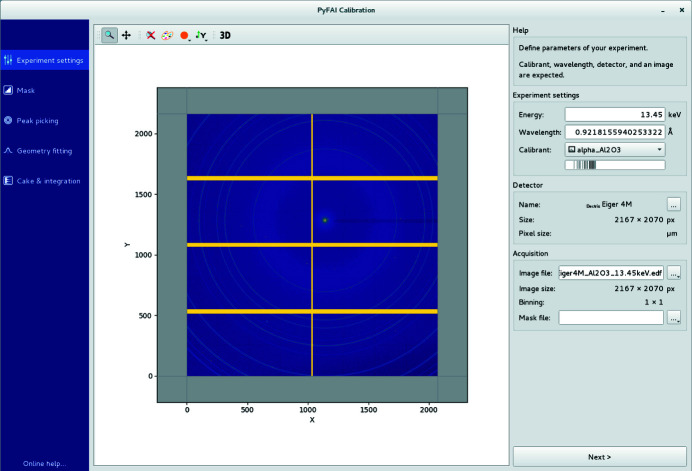
The experiment settings tab is used to load the calibration image, set the energy, the calibrant and select the detector for the subsequent analysis. The binning mode of the detector is automatically guessed.

**Figure 3:**
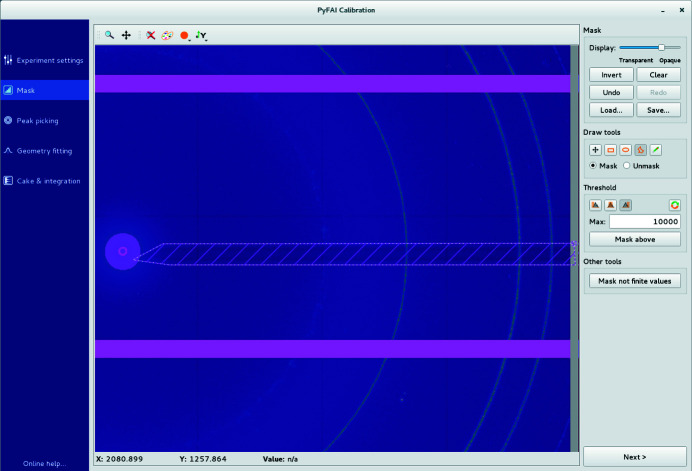
The mask-drawing tool is used to exclude pixels using a rectangular, polygonal or pencil selection. Pixels can also be selected according to their value (thresholding).

**Figure 4:**
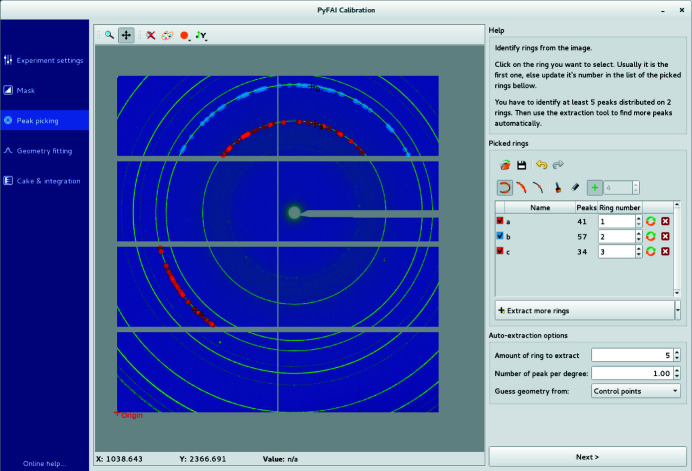
The peak-picking tool automatically selects a group of contiguous local maxima in the image close to the clicked peak which then needs to be assigned to the correct calibrant ring number in the right-hand side panel.

**Figure 5:**
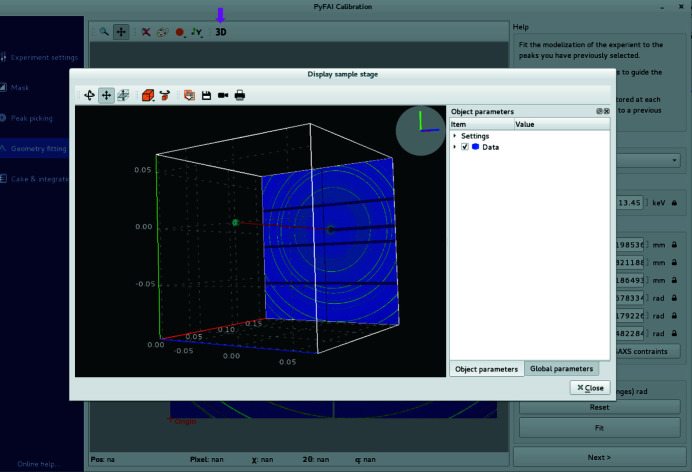
In the geometry fitting tab, each variable can be fixed or left free for refinement within a given range. A 3D representation of the experimental setup allows visualization of the relative position of the sample, direct beam and detector after fitting.

Following the survey conducted among the beamline scientists of the ESRF, a new graphical user interface (GUI) design was developed on top of *pyFAI*. This new GUI, called *pyFAI-calib2*, is based on the *silx* scientific widgets (Knobel *et al.*, 2017 ▸), itself based on the *PyQt5* library (Thompson, 2013 ▸). The *pyFAI-calib2* tool has been specifically designed to address the needs of novice users, but should perform equally well regardless to the scientific field or experimental setup. The calibration of the experimental setup based on Debye–Scherrer rings is carried out in five steps and presented as a software wizard with five subsequent tabs within the graphical interface:

(i) Experimental settings. This first tab lets the user select the wavelength (or the energy), the reference material for calibration (calibrant) and choose the type of detector, either from a list of 50 provided, or by defining a new detector (Fig. 2 ▸).

(ii) Mask drawing. This tab allows us to mask-out unwanted regions/pixels from the image, either based on their intensity (thresholding) or simply by drawing polygons on the image. Different masks can be saved, retrieved and assembled (Fig. 3 ▸).

(iii) Peak picking. Individual peaks and arcs of rings can be segmented out (automatically) and assigned (manually) to different rings, each associated with a single reflection of the calibrant (Fig. 4 ▸).

(iv) Geometry fitting. At this stage, the detector position and wavelength are fitted against peak positions and ring numbers. Any of the parameters can be fixed or let free for refinement, possibly with boundaries. The (calculated) positions of the beam centre and the PONI, and the expected positions of rings are overlaid onto the diffraction image to visually assess the quality of the fit. The sample, detector and direct beam can also be visualized in 3D (Fig. 5 ▸).

**Figure 6:**
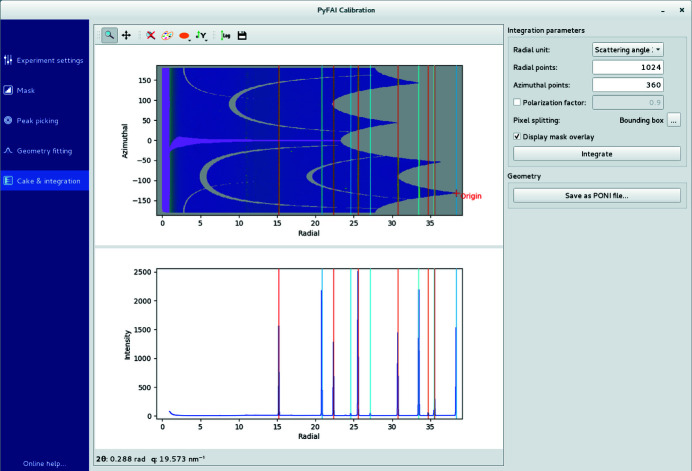
The cake and integration tab displays the 1D curve and 2D integrated image with the calibrant ring positions overlaid to allow easy validation of the quality of the calibration.

(v) Integration. This tab displays the 1D and 2D integrated patterns (*i.e.* powder diffraction profile and caked-image) to further validate the modelling of the experimental setup (Fig. 6 ▸). The algorithm used for integration, the pixel splitting scheme and the radial unit can also be changed in this tab. Diffraction profiles can be saved as text files or images as well as the experimental setup description file (.poni file) for subsequent use with other tools from the *pyFAI* suite.

The online documentation of *pyFAI* contains a quick tutorial (Valls & Kieffer, 2019 ▸) designed for novice users to provide them with their scattering geometry in a couple of minutes. Diffraction images can be provided in HDF5 format (Collette, 2013 ▸) or any of the dozens of file formats supported by the *FabIO* library (Knudsen *et al.*, 2013 ▸).

## 5. Calibration of a detector on a moving stage   

Calibration of a detector mounted on a translation stage along the direct beam has successfully been exploited by *DAWN* (Filik *et al.*, 2017 ▸) and *GSAS-II* (Horn *et al.*, 2019 ▸) to remove the correlation between the detector distance and the energy of the beam. While these two software packages offer an intuitive user interface for those experiments, the modelization of the translation stage remains hard-coded. This section explains how to describe, programmatically, a moving stage in *pyFAI*, calibrate the proposed model and use it to perform the azimuthal integration with many images.

The calibration of the detector position on a fixed goniometer position can be performed with *pyFAI* as long as several rings of the chosen calibrant are present in the image and that five control points can be extracted from one ring and at least one point from another ring. Of course, more points provide a better fit but this limit of two visible rings may be an issue when very small area detectors are used (*e.g.* the ImXPAD S10).

### 5.1. Transformation of geometry   

Here, the difficulty is not in the calibration, but rather in the variety of goniometers and translation stages which may be controlled by one or multiple motors. The goniometer description implemented in *pyFAI* accepts an arbitrary number of motors attached to the goniometer/translation stage. Let *motors* be a list of *n* motors moved during the acquisition: (*motor*_1_, *motor*_2_,…, *motor*_*n*_).

Calibration of the goniometer involves defining a model and optimizing it:

(i) Choose a list of *m* static parameters for the model which are used to describe the position of the detector, *m* being the number of DoFs of the model: *params* = (*param*_1_, *param*_2_,…, *param*_*m*_).

(ii) Define the six *transformation functions* 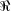 which transform the motor positions (*motors*) and goniometer parameters (*params*) into the six PONI parameters used to initialize an azimuthal integrator in *pyFAI*:
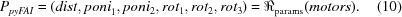



(iii) Optimize the parameter set of the goniometer (params) so that, for any position of the motors, the detector position is expressed as a PONI-parameters set.

The six PONI parameters returned are then used to calculate the experimentally observed 2θ_exp_ position of the peaks over all control points. Those 2θ_exp_ are compared with the theoretically expected 2θ_theo_ values calculated from the calibrant *d*-spacing and the wavelength,
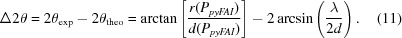
The average of the squares of these differences is used as a cost function to optimize the parameters using the *scipy.optimize.minimize* function from *SciPy* (Jones *et al.*, 2001 ▸),
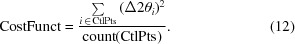
From a computer engineering perspective, these *transformation functions* present several contradictory challenges: the user should be able to express any numerical transformation (flexibilty), but should be prevented from executing any arbitrary code (security). Moreover, those functions should be made persistable on disk (*i.e.* saved) and their restoration should be possible without having to re-perform the calibration, nor creating vulnerabilities.

The *NumExpr* library (Cooke *et al.*, 2009–2017 ▸) allows the evaluation of textual mathematical formulae into numerical functions, offering an arbitrary number of parameters for the goniometer definition and as many motors as needed. *NumExpr*, being a mathematical compiler, cannot execute other types of code. The mathematical expressions provided by the user are simply saved together with the motor names and parameter names and values in a JSON-format (Crockford, 2017 ▸) on the disk.

In the following example, the definition of those functions is simply the creation of an object from the *GeometryTransformation* class (*i.e. instantiation*), with a list of motor names (*pos_names*), parameter names (*param_names*), as well as a set of six formulae, one for each of the six PONI parameters.

### 5.2. A simple example: the translation table   

At the ESRF, the protein crystallography beamlines use large area pixel detectors (typically Pilatus 6M from Dectris) placed on a translation table which allows collection of the data at the optimal distance: shorter sample-to-detector distances to explore the region for high-*q* or longer distances for better Bragg peak separation.

The *MX-calibrate* tool from the *pyFAI* suite is available for the calibration of many images taken at various distances. This can also be interpreted in terms of the goniometer setup (actually a translation stage), where the sample-to-detector distance is modelled as a simple linear function of the table position:[Chem scheme1] 
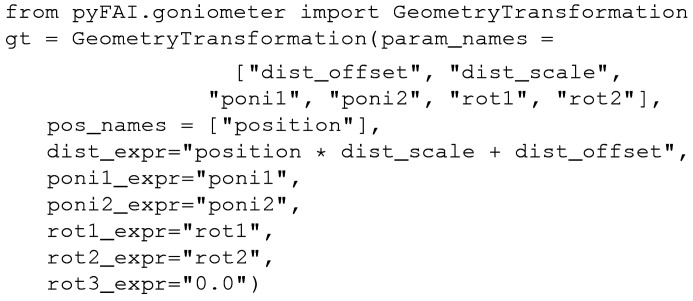


In the example above, a six-parameter model was chosen for the goniometer geometry, most parameters matching exactly those of *pyFAI*: *poni*_1_, *poni*_2_, *rot*_1_ and *rot*_2_. *rot*_3_ is forced to zero while the distance is defined as a linear function of the motor position.

**Figure 7:**
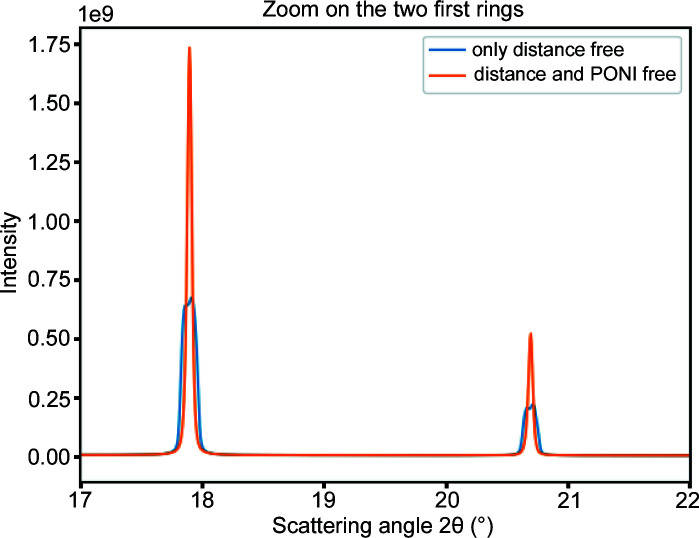
Powder diffraction profile obtained from seven images acquired at various distances from 15 cm to 45 cm. The translation table position is combined with six (resp. eight) parameters model-fitted, where *dist* (resp. *dist*, *poni*_1_ and *poni*_2_) depends linearly on the table position.

The content of this tutorial is available as a *Jupyter notebook* (Pérez & Granger, 2007 ▸) and forms part of the official documentation of *pyFAI* (Kieffer & Flot, 2017 ▸); it is directly visible in a web-browser. In addition, one can replay the notebook with *Jupyter* as it is self-consistent: all images are automatically downloaded and cells can be modified or adapted. The notebook presents the usage of the geometry transformation class together with the translation table refinement. Initially, this set of data was calibrated using the ‘MX-calibrate’ tool which automatically extracts the control points from images taken at the ESRF ID29 beamline (energy 12.75 keV) from the metadata written in the image headers, thus a set of control points is available prior to data processing. The translation table is calibrated using all control points and this geometry transformation object. As a result, all images have been integrated azimuthally. Fig. 7 ▸ shows the first two Bragg peaks of CeO_2_ calibrated using this methodology. The blue curve corresponds to this initial model refined; the two peaks look ‘doubled’, indicating a poor modelling of the geometry.

To address this poor modelling, another transformation function is defined, with a few additional DoFs on the PONI position (hypothesis: the translation table is not perfectly parallel to the incident beam). Once three parameters (*dist*, *poni*_1_ and *poni*_2_) have been re-fitted and all data integrated with the new model, one obtains the orange curve in Fig. 7 ▸. Its peaks are much sharper and the residual cost is about five times smaller, indicating a much better fit.

This example shows that the PONI is moving on the detector plane by 1‰ horizontally and 4‰ vertically. This ‘large’ vertical deviation has been confirmed by the beamline staff and is related to the last focusing mirror placed just before the sample, causing the beam to deflect away from the horizontal. Once everything is fitted, the quality of the geometry obtained is perfectly suited to powder diffraction experiments for any detector position.

In this example, all motor positions used for calibration were within the accessible range, hence positions have been interpolated. The next example will validate that extrapolation outside the calibration range is also possible.

### 5.3. A more realistic example: the single-axis goniometer   

Probably the main application of this work is to place a small area detector on a goniometer 2θ arm in replacement of a punctual detector (*e.g.* diode) for powder diffraction measurements. The initial idea was to use a small region of interest (ROI) in the centre of an area detector, and check whether it was possible to rebuild a powder diffraction pattern when moving the detector at various goniometer angles. This ROI has to be of limited size in the dimension orthogonal to the ring and as extended as possible in the tangential direction, with the limit of the curvature of the ring (especially at low 2θ angles). We considered an ROI 10 pixels wide within a Pilatus 100k (487 pixels × 195 pixels) which means using only 2% of the total width of the detector (different lines being used for different bins). By using the full detector area, the signal/noise ratio could be improved by a factor of seven [(487/10)^1/2^] if the movement of the detector on the goniometer is perfectly predictable and reproducible.

When the detector arm is moving in the vertical plane (around an horizontal axis), a simple *geometry transformation* is defined with *rot*_2_ (in radians) as a linear transformation of the motor position (*pos* is the default motor name), here measured in degrees by the goniometer. The scale parameter, scale = π/180, is used to convert angular units. The other parameters *dist*, *poni*_1_, *poni*_2_ and *rot*_1_ are directly mapped to *pyFAI*’s parameters. As done previously, *rot*_3_ is kept fixed at zero:[Chem scheme2] 
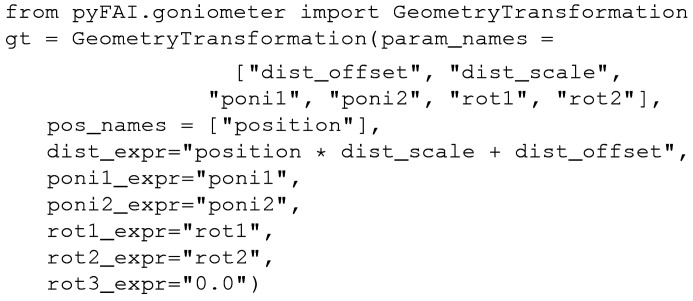


**Figure 8:**
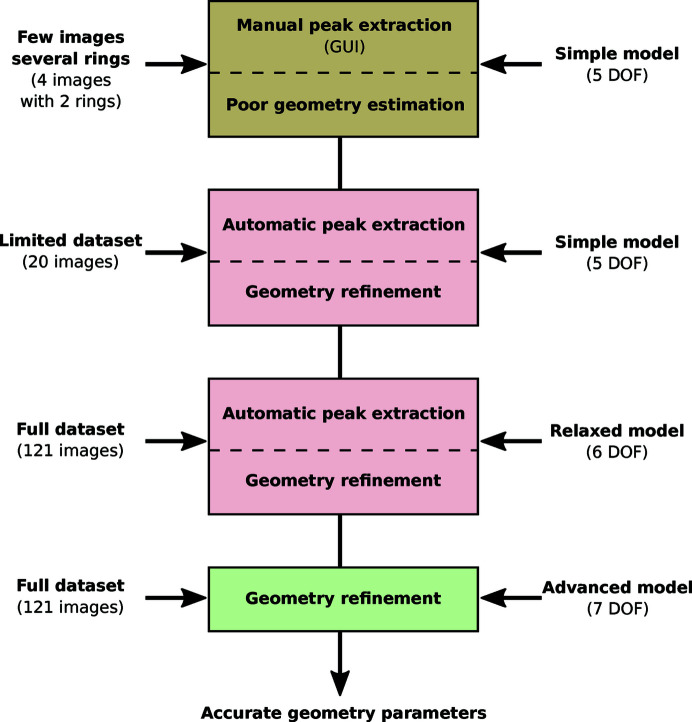
Workflow used to calibrate a set of 121 images collected with a Pilatus 100k mounted on a moving 2θ arm and detailed in the *Jupyter Notebook* given by Kieffer & Hennig (2017 ▸).

This example is also available in a *Jupyter notebook* (Kieffer & Hennig, 2017 ▸) and the workflow used is depicted in Fig. 8 ▸. This experiment has been performed using a single-module Pilatus (100k) detector which is mounted on a 2θ arm, moving from 5° to 65° in 0.5° steps on the ROBL beamline (Kvashnina & Scheinost, 2016 ▸), ESRF BM20. In this experiment, 121 diffraction images of LaB_6_ reference compound material were acquired. Due to the small size of the detector, some of the images present no rings at all (especially at low 2θ angles), most have only one ring and only a few images display two rings. Even if those images are technically suitable for static detector calibration, they still present a challenge due to the large diffraction angles (2θ > 30°), the low curvature of the rings and the difficulty in assigning the picked peaks with the proper calibrant ring number.

Four images have been manually calibrated, fixing the vertical rotation axis of the detector (*rot*_1_ = 0) to guarantee some consistency between the calibrations.

**Figure 9:**
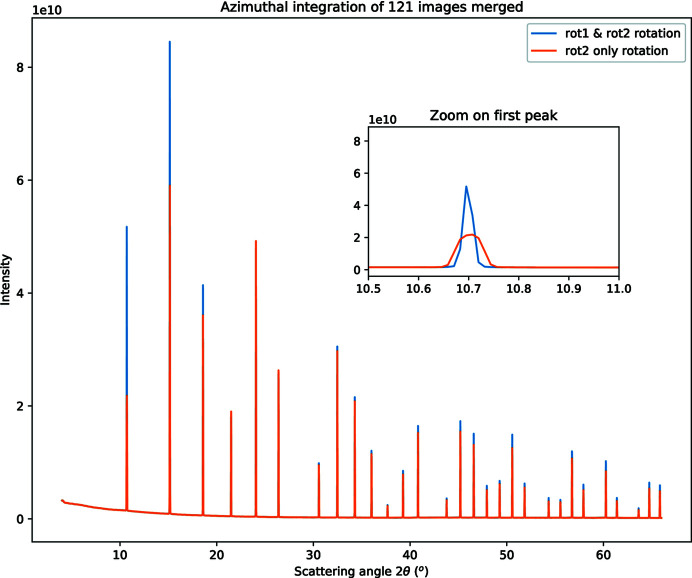
Powder diffraction pattern obtained from 121 Pilatus 100k images acquired at goniometer angles ranging from 5° to 65° on an LaB6 reference sample at 16 keV. The insert is a close-up view of the first peak showing the sharpness of the signal depending on the model. The orange curve corresponds to the simple model where *rot*_2_ depends linearly on the goniometer angle (six DoFs). The blue curve corresponds to an advanced model where both rotations (*rot*_1_ and *rot*_2_) depend linearly on the goniometer angle (seven DoFs).

An initial simple model with a set of parameters where *rot*_2_ is equal to the goniometer angle has first led to convergence with some constraints and bounds. In a second step, 20 images in the vicinity of the first set were added to the model, their peaks extracted according to the initial model and the model refined using the control points from the 20 images. Then, all other images were added to the model, and additional control points were automatically extracted from all images according to the previous geometry model. Finally, all constraints and bounds were removed, and the model was refined again and used to generate a *MultiGeometry* object, which is suitable for integrating many images together. After integration of all 121 images, the powder diffraction pattern displayed in Fig. 9 ▸ is obtained (orange curve). All peaks of the curve appear at the correct scattering angle but the first few peaks look exceedingly broad.

**Figure 10:**
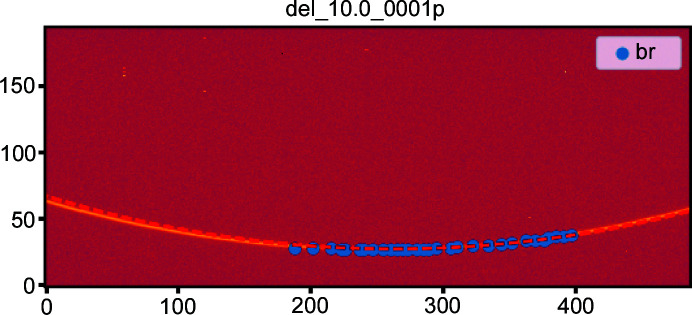
Diffraction image taken with the goniometer arm at 10°. The control points are in blue and the expected ring of the simple model [*rot*_2_ = *f*(*pos*)] is the dashed red line. This highlights the need for *rot*_1_ to depend on the goniometer position.

This broadening is confirmed by looking at the first ring’s image, where the goniometer angle was set to 10° (Fig. 10 ▸). The expected position of the ring (dashed red line) does not properly correspond to the actual ring (yellow on the image). The control points extracted and used in the fitting are plotted in blue.

The fitting procedure averaged the *rot*_1_ value (rotation around the vertical axis) over all images, but Fig. 10 ▸, which is taken on the first ring, suggests this value is slightly wrong. The images of the last rings (visible in the notebook) indicate a small offset but in the other direction. The fact that both shifts are small suggests to use a first-order correction on *rot*_1_. A new model was tested where both the *rot*_1_ and *rot*_2_ values are scaled with the motor position. After refinement, the cost function dropped by a factor two and the low-angle peaks became sharp (blue curve in Fig. 9 ▸).

This parameter set was saved and allowed a few other compounds to be analysed and compared with the same pattern recorded with the Pilatus being considered as a point detector. The signal/noise ratio was found to be much better with an acquisition time 24 times faster (10 min instead of 4 h). The average peak resolution full width at half-maximum FWHM = 0.02° obtained on LaB_6_ (see notebook) is a factor of ten worse than the highest achieved resolution with secondary monochromator on insertion devices (Dejoie *et al.*, 2018 ▸), but such an experiment requires 24 h acquisition.

A third example of goniometer calibration is available in the work by Kieffer & Blanc (2017 ▸). It corresponds to the calibration of an ImXPAD detector (Boudet *et al.*, 2003 ▸) composed of eight stripes of seven modules, many of which are defective. This detector is mounted on the goniometer arm at the D2AM beamline (Ferrer *et al.*, 1998 ▸), ESRF BM02. This example is conceptually the same as that from the ROBL beamline, with a few differences: (i) all images are fitted directly with eight DoFs; (ii) the detector is larger, hence the calculation time is longer, especially when it comes to ring extraction; (iii) the mask needs some extra care to remove a few hot pixels; the detector is mounted, rotated by 90° on the arm, thus *rot*_3_ = π/2.

This generic method has even been extended to strip detectors like the Mythen detector manufactured by Dectris as shown in the tutorial on the calibration of the array of nine Mythen detectors (Kieffer & Picca, 2018 ▸) mounted on the goniometer arm of beamline Cristal at Synchrotron Soleil.

## 6. Outlook   

The goniometer description in this work can be adapted to many types of goniometers. The transformation function class presented in this manuscript may be extended in the future to use *libhkl* (Picca, 2010–2016 ▸), which already contains many diffractometer geometries with their associated rotation matrices.

Different generations of pixel detectors have seen their pixel sizes shrinking: from 172 µm for Pilatus, 130 µm for ImXPAD, 75 µm for Eiger and 55 µm for Medipix-based chips, and probably even less for future-generation detectors dedicated to coherent diffraction imaging. As the resolution of the powder diffraction pattern obtained is often limited by the pixel size (and the sample-to-detector distance), this shrinkage of pixel sizes naturally leads to higher quality powder diffraction patterns. Unfortunately, to keep the covered 2θ range constant, one would need to multiply the number of pixels by the square of the pixel size reduction factor, and the associated infrastructure for read-out and data transfer accordingly. Moving the detector offers a flexibility which removes this limitation but makes the experiment slightly slower – though still suitable for *in situ* experiments.

## 7. Conclusions   

The new graphical user interface of *pyFAI* has been developed to ease the calibration of an experimental setup with static detectors, especially for novice users. The concept of calibration of the detector position has been extended to fit the detector position as a function of the motion of a goniometer. Once a few fixed positions of the goniometer have been calibrated, a model can be optimized and the detector position can be extrapolated at any goniometer configuration. By acquiring multiple images at various positions, these images can be integrated together to produce a high-*q* powder diffraction pattern of quality equivalent to that acquired with a much larger detector, opening up new opportunities for *in* *situ* experiments and PDF measurements on most synchrotron diffraction beamlines.
